# Bleeding risk of ticagrelor compared to clopidogrel in intensive care unit patients with acute coronary syndrome: A propensity-score matching analysis

**DOI:** 10.1371/journal.pone.0232768

**Published:** 2020-05-04

**Authors:** Thibault Charpentier, Cyril Ferdynus, Thomas Lair, Charlotte Cordier, Caroline Brulliard, Dorothée Valance, Malo Emery, Margot Caron, Nicolas Allou, Jérôme Allyn

**Affiliations:** 1 Réanimation Polyvalente, Centre hospitalier universitaire Félix Guyon, La Réunion, Saint-Denis, France; 2 Unité de Soutien Méthodologique, Centre hospitalier universitaire Félix Guyon, La Réunion, Saint-Denis, France; 3 INSERM, CIC 1410, Saint-Pierre, France; 4 Département d’Informatique Clinique, Centre hospitalier universitaire Félix Guyon, La Réunion, Saint-Denis, France; Erasmus Medical Center, NETHERLANDS

## Abstract

**Background:**

Intensive care unit (ICU) patients with the most severe forms of acute coronary syndrome (ACS) require invasive therapies such as extracorporeal life support. The risk of bleeding in ICU patients with ACS treated with a dual antiplatelet therapy of aspirin and ticagrelor is unknown. The primary objective of this study was to compare the bleeding risk of ticagrelor and clopidogrel in ICU patients with ACS.

**Methods and findings:**

We conducted a retrospective study based on a propensity score and a proportional hazards model. All patients with ACS hospitalized in the ICU of a French university hospital between January 2013 and January 2017 were included in the study. Bleeding during ICU stay was defined as all Thrombolysis in myocardial infarction (TIMI) major or minor events. A total of 155 patients were included in the study. According to propensity score matching, 57 patients treated with aspirin and ticagrelor were matched with 57 patients treated with aspirin and clopidogrel. Median (first-third quartile) Simplified Acute Physiology Score II was 61.5 (41.0–85.0). Bleeding during ICU stay occurred in 12 patients (21.1%) treated with clopidogrel and in 35 patients (61.4%) treated with ticagrelor (*p*<0.0001). This significant association was found for both TIMI major bleeding (12.3% *vs*. 35.1%, *p* = 0.004) and TIMI minor bleeding (8.8% *vs*. 26.3%, *p* = 0.01). The relative risk of bleeding occurrence during ICU stay was 2.60 (confidence interval 95%: 1.55–4.35) for ticagrelor compared to clopidogrel. No significant difference in ICU mortality was found between the two groups (45.6% in the clopidogrel group *vs*. 29.8% in the ticagrelor group, *p* = 0.08).

**Conclusions:**

Bleeding complications are frequent and serious in ICU patients with ACS. A dual antiplatelet therapy of aspirin and ticagrelor is associated with a higher risk of bleeding compared to a dual antiplatelet therapy of aspirin and clopidogrel.

## Background

In addition to emergency coronary revascularization *via* percutaneous coronary intervention (PCI) or cardiac surgery, treatment of acute coronary syndrome (ACS) includes dual antiplatelet therapy in which aspirin is combined with a P2Y12 receptor inhibitor (clopidogrel, prasugrel, or ticagrelor). For several years now, ticagrelor has been recommended over clopidogrel in the absence of contraindications, due to greater efficacy and speed of action, even though an increased risk of bleeding has been reported in several cardiology studies [1–4].

The most severe cases of ACS require intensive care management, especially in the presence of shock and/or severe respiratory distress. This is an important issue given that the percentage of ACS patients with Killip class IV (*i*.*e*. cardiogenic shock) reported in the literature is significant, ranging from 1 to 12% [5–7]. In fact, intensive care management of critically ill ACS patients often requires invasive techniques that can cause bleeding (central venous catheter, arterial catheter, mechanical ventilation, renal replacement therapy, circulatory assistance, etc.). To our knowledge, no study to date has examined the risk of bleeding in Intensive Care Unit (ICU) patients with ACS treated with ticagrelor. The primary objective of this study was to compare the bleeding risk of ticagrelor and clopidogrel in ICU patients diagnosed with ACS.

## Methods

This retrospective study was approved by the Institutional Review Board of the Ethics Committee of Reunion Island University Hospital (reference R15007). The need for informed consent was waived because of the observational and retrospective nature of the study. This article follows the STROBE reporting guidelines for observational studies [8,9].

### Study population

We conducted a retrospective observational study in a 23-bed medical-surgical adult ICU in a French university hospital. This university hospital is the regional reference center for the management of patients with ACS and/or cardiogenic shock. It includes a technical center open 24/7, which offers coronary angiography, extracorporeal life support (ECLS), and cardiac surgery services. As the study was designed following the introduction of ticagrelor in our institution in 2012, the inclusion period was from January 2013 to January 2017. We retrospectively analyzed the medical records of all patients with ACS who were hospitalized in our ICU during the inclusion period. Exclusion criteria were: age below 18 years, treatment of ACS with cardiac surgery, treatment of ACS with thrombolysis and/or prasugrel, and incomplete/lost medical record. Patients could be included in the study only once, and the analysis focused on the first ACS-related stay in ICU.

During the study period, a regional protocol for the management of patients with ACS in the emergency department or outside hospital was designed based on European recommendations [10]. Accordingly, the treatment of STEMI, administered prior to PCI, was composed of: (*i*) intravenous aspirin (250 mg) and (*ii*) ticagrelor (180 mg) or clopidogrel (600 mg), the former being preferred in the absence of contraindications (*i*.*e*., history of hemorrhagic stroke, severe hepatic insufficiency, chronic hemodialysis, ongoing anticoagulant treatment) and (*iii*) intravenous enoxaparin (0.5 mg.kg^-1^) or, in cases of severe chronic renal failure, unfractionated heparin (bolus of 70 UI.kg^-1^, and then 12 UI.kg^-1^.h^-1^). Thrombolysis was administered when PCI could not be performed within 120 minutes of ACS onset. Ticagrelor and clopidogrel were administered in ICU at a dose of 90 mg twice daily and 75 mg daily, respectively.

### Data collection and processing

Demographic data, test results, therapeutics used, and patient progress before and during ICU stay were analyzed. Time of inclusion was defined by the date of initiation of treatment with clopidogrel or ticagrelor. The primary outcome was bleeding occurrence during ICU stay according to the Thrombolysis in Myocardial Infarction (TIMI) criteria which have been used in most cardiovascular trials and recommendations for the past thirty years [2,4,5,10,11].

Bleeding was classified as TIMI major or minor; we did not record the TIMI minimal. In case of multiple bleeding in the same patient, the most severe TIMI bleeding event was recorded. The secondary outcomes were ICU mortality, ICU length of stay, and other markers of bleeding such as blood transfusion.

### Definitions

ST-segment elevation myocardial infarction (STEMI), non-ST-segment elevation myocardial infarction (NSTEMI), and unstable angina were defined according to the European Society of Cardiology guidelines for the management of ACS [12].

Cardiogenic shock was defined by: systolic blood pressure < 90 mm Hg for more than 30 minutes, need for vasopressors to achieve systolic blood pressure > 90 mm Hg, pulmonary congestion, elevated left ventricular filling pressure (*i*.*e*. pulmonary capillary wedge pressure > 18 mmHg), reduced cardiac index (< 1.8 L.min^-1^.m^-2^ without support and 2.0–2.2 L.min^-1^.m^-2^ with support), and/or signs of impaired organ perfusion as manifested by one of the following: *i*) altered mental status, *ii*) cold, clammy skin and extremities, *iii*) oliguria with urine output < 30 mL.hour^-1^, *iv*) serum lactate > 2.0 mmol.L^-1^ [13].

In accordance with the TIMI classification, major bleeding was defined as: *i*) any intracranial bleeding (excluding microhemorrhages <10 mm evident only on gradient-echo magnetic resonance imaging) or *ii*) clinically overt signs of hemorrhage associated with a drop in hemoglobin of ≥ 5 g.dL^-1^ or a 15% absolute decrease in hematocrit or *iii*) fatal bleeding (bleeding that directly results in death within 7 days). Minor bleeding was defined as: *i*) any clinically overt sign of hemorrhage (including imaging) associated with a drop in hemoglobin of 3 to < 5 g.dL^-1^ or a 10% to 15% decrease in hematocrit or *ii*) no observed blood loss, *i*.*e*., a drop in hemoglobin of 4 g.dL^-1^ to < 5 g.dL^-1^ or a 12% to 15% decrease in hematocrit. Minimal bleeding was any overt sign of hemorrhage that did not meet the criteria for major or minor bleeding defined above and that met one of the following criteria: *i*) requiring intervention (medical practitioner-guided medical or surgical treatment to stop or treat bleeding, including temporarily or permanently discontinuing or changing the dose of a medication or study drug), *ii*) leading to or prolonging hospitalization, *iii*) prompting evaluation (leading to unscheduled visit to a healthcare professional and diagnostic testing, either laboratory or imaging) [14].

### Statistical analysis

Results were expressed as frequencies and percentages for categorical variables, and as median and interquartile range for continuous variables. Categorical variables were compared using chi-square test or Fisher’s exact test, as appropriate. Continuous variables were compared using Student’s t-test or Mann-Whitney U test, as appropriate.

### Propensity score

Propensity score was defined as the probability of exposure to ticagrelor. To limit over adjustment which might result from using this score, we selected only the covariates likely to introduce a confounding bias [15,16].

The propensity score was estimated using a logistic regression adjusted for age, gender, place of initial care, history of chronic renal failure and/or hypertension and/or heart failure, and usual treatment with clopidogrel and/or ticagrelor and/or aspirin. Matching was then performed between patients exposed to ticagrelor and unexposed patients with a propensity score caliper of 0.05. After propensity score matching, standardized differences were estimated to compare baseline characteristics and, therefore, to assess the accuracy of the matching procedure.

### Analysis of outcomes

Cumulative incidence of bleeding was modeled using a proportional hazards model for the subdistribution of a competing risk, for clustered data [17,18]. Death was the only competing risk considered. The proportional hazards model was stratified by group of patients defined by propensity score matching. Hazard ratios and their 95% confidence intervals (CI) were calculated. A two-tailed *p* value < 0.05 was considered significant.

Statistical analyses were performed using SAS 9.4 (SAS Institute, Cary, NC, USA) and R (package crrSC v1.1).

## Results

**Fig 1:**
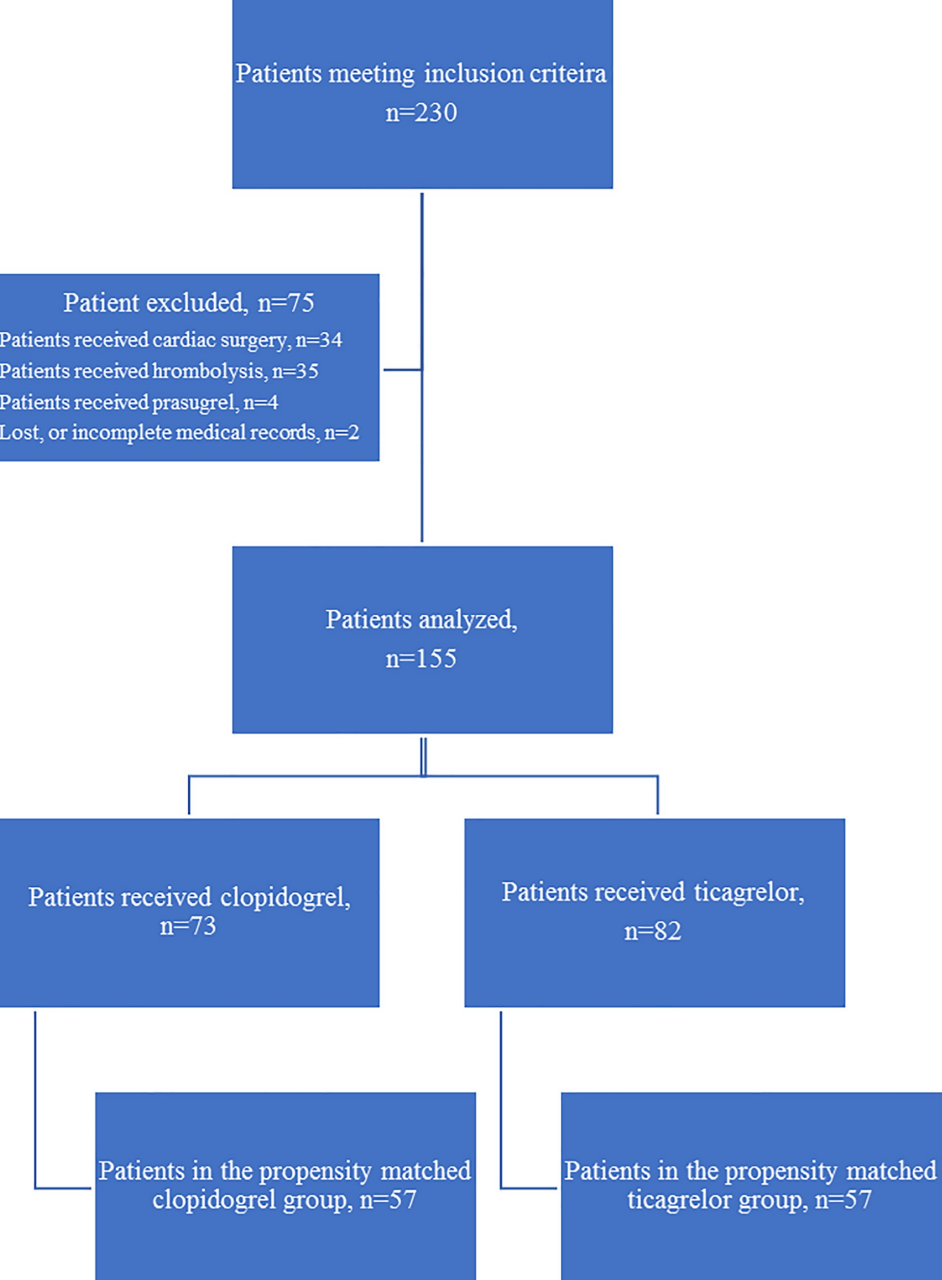
Flow chart of the study, illustrating the matched groups according to propensity score.

Over the study period, 230 patients were hospitalized in ICU with a diagnosis of ACS. Of these, 75 were excluded from the study (35 patients received thrombolysis, 34 patients received cardiac surgery, 4 patients received prasugrel, and 2 patients had incomplete or lost medical records). A total of 155 patients were included in the study. Using propensity score matching, 57 patients were included in the clopidogrel group and 57 were included in the ticagrelor group **(Fig 1**).

### Characteristics of the 155 included patients (before matching)

The characteristics of the 155 included patients at study inclusion and between study inclusion and ICU discharge or death are compared in S1 Table and S2 Table. Median age was 65.0 (55.8–73.0) years old. During the study period, all patients were treated with aspirin, 82 patients (52.9%) were treated with ticagrelor and 73 (47.1%) were treated with clopidogrel. Comparison of patients treated with ticagrelor and patients treated with clopidogrel at study inclusion showed mainly differences in age, presence of hypertension or chronic renal failure, and usual treatment with clopidogrel Significant differences in SAPS II, renal failure, and initial treatment were observed between study inclusion and ICU discharge or death.

### Characteristics of the 114 matched patients

According to propensity score matching, 57 patients treated with ticagrelor were matched with 57 patients treated with clopidogrel.

No significant differences in patient characteristics at study inclusion were found between the ticagrelor group and the clopidogrel group, as shown in **Table 1**.

**Table 1:** Characteristics of the 114 matched patients at study inclusion.

Variables	All patients (*n* = 114)	Clopidogrel (*n* = 57)	Ticagrelor (*n* = 57)	Standardized difference
Age (years)	65.4 (56.1–73.0)	65.8 (56.4–73.1)	65.1 (56.1–71.9)	0.00
Male gender	83 (72.8%)	42 (73.7%)	41 (71.9%)	-0.04
History of				
Hypertension	75 (65.8%)	40 (70.2%)	35 (61.4%)	-0.18
Diabetes mellitus	66 (57.9%)	35 (61.4%)	31 (54.4%)	-0.14
Smoking (current or former)	64 (56.1%)	28 (49.1%)	36 (63.2%)	0.28
Dyslipidemia	39 (34.2%)	21 (36.8%)	18 (31.6%)	-0.11
Chronic heart failure	35 (30.7%)	19 (33.3%)	16 (28.1%)	-0.11
Coronary artery disease	31 (27.2%)	16 (28.1%)	15 (26.3%)	-0.04
Peripheral artery occlusive disease	18 (15.8%)	9 (15.8%)	9 (15.8%)	0.00
Chronic renal failure	14 (12.3%)	7 (12.3%)	7 (12.3%)	0.00
Ischemic stroke	11 (9.6%)	7 (12.3%)	4 (7.0%)	-0.18
Body mass index > 30kg.m^-2^	11 (9.6%)	7 (12.3%)	4 (7.0%)	-0.18
Sleep apnea syndrome	8 (7.0%)	3 (5.3%)	5 (8.8%)	0.14
Cancer	7 (6.1%)	4 (7.0%)	3 (5.3%)	-0.07
Chronic obstructive pulmonary disease	7 (6.1%)	3 (5.3%)	4 (7.0%)	0.07
Hazardous alcohol use	2 (1.8%)	1 (1.8%)	1 (1.8%)	0.00
Usual treatment				
Oral anticoagulant	3 (2.6%)	2 (3.5%)	1 (1.8%)	-0.11
Aspirin	44 (38.6%)	22 (38.6%)	22 (38.6%)	0.00
Clopidogrel	19 (16.7%)	11 (19.3%)	8 (14.0%)	-0.14
Ticagrelor	2 (1.8%)	1 (1.8%)	1 (1.8%)	0.00
Place of initial care				-0.13
Outside hospital	68 (59.6%)	32 (56.1%)	36 (63.2%)	0.14
In hospital, emergency department	24 (21.1%)	13 (22.8%)	11 (19.3%)	
In hospital, other department	22 (19.3%)	12 (21.1%)	10 (17.5%)	
Cardiac arrest before ICU admission	53 (46.5%)	28 (49.1%)	25 (43.9%)	-0.10
Type of ACS : STEMI*	88 (77.2%)	42 (73.7%)	46 (80.7%)	0.17

Results are expressed as median (first and third quartile) and numbers (proportions). ACS: Acute coronary syndrome; ICU: Intensive Care Unit; STEMI: ST-segment elevation myocardial infarction.

*opposite to “non-ST-segment elevation myocardial infarction”.

The characteristics of matched patients treated with ticagrelor or clopidogrel between study inclusion and ICU discharge or death are compared in **Table 2**. Patients treated with ticagrelor had a significantly lower SAPS II than patients treated with clopidogrel (60.0 (38.0–80.0) *vs*. 69.0 (46.0–88.0), *p* = 0.03). While there were no significant differences in dobutamine or norepinephrine use between the two groups, epinephrine use in the first 24 hours after admission to ICU was significantly rarer in the ticagrelor group than in the clopidogrel group (14% *vs*. 31.6%, *p* = 0.03). Coronary angiography was performed in 108 patients (94.7%), and ≥1 coronary stent(s) were implanted in 90 patients (83.3%). Glycoprotein IIb/IIIa inhibitors (tirofiban) were used in 36 patients (31.6%), with no significant difference between the two groups. No significant differences in specific treatment for ACS were observed between the two groups. Creatinine levels, alanine and aspartate aminotransferase, prothrombin, and activated partial thromboplastin time at ICU admission were abnormal in both groups with no significant differences between the two groups. No significant differences in biological and clinical parameters and other treatments were found between the two groups.

**Table 2:** Characteristics and outcomes of the 114 matched patients between study inclusion and ICU discharge or death.

Variables	All patients (*n* = 114)	Clopidogrel (*n* = 57)	Ticagrelor (*n* = 57)	*P value*
Coronary angiography	108 (94.7%)	52 (91.2%)	56 (98.2%)	0.21
Stent	90 (83.3%)	43 (82.7%)	47 (83.9%)	0.86
ECLS during coronary angiography	5 (4.4%)	3 (5.3%)	2 (3.5%)	1.00
Emergency care initiated for ACS				
Glycoprotein IIb/IIIa inhibitors (tirofiban)	36 (31.6%)	14 (24.6%)	22 (38.6%)	0.11
Aspirin	114 (100.0%)	57 (100.0%)	57 (100.0%)	NA
Low Molecular Weight Heparin	94 (82.5%)	45 (78.9%)	49 (85.9%)	0.32
Unfractionated Heparin	20 (17.5%)	12 (21.0%)	8 (14.0%)	0.32
At ICU admission				
Simplified Acute Physiology Score II	61.5 (41.0–85.0)	69.0 (46.0–88.0)	60.0 (38.0–80.0)	0.03
Therapeutic hypothermia	1 (0.9%)	0 (0.0%)	1 (1.8%)	1.00
Left ventricular ejection fraction	0.3 (0.2–0.5)	0.3 (0.2–0.4)	0.4 (0.3–0.5)	0.11
Biological parameters at ICU admission				
Hemoglobin level (g.dL^-1^)	12.3 (10.5–13.7)	12.4 (10.5–13.7)	12.2 (10.6–13.7)	0.89
Platelet count (G.L^-1^)	227.0 (188.0–274.0)	219.5 (171.0–265.0)	239.0 (207.0–276.0)	0.29
aPTT (ratio)	1.6 (1.3–3.5)	1.5 (1.3–3.0)	2.1 (1.3–3.5)	0.95
Prothrombin (%)	68.0 (55.0–76.0)	67.0 (55.0–74.0)	69.5 (56.0–76.0)	0.40
Urea (mmol.L^-1^)	7.9 (5.2–13.2)	9.3 (5.9–15.0)	7.2 (4.8–10.6)	0.34
Creatinine (μmol.L^-1^)	123.0 (86.0–212.0)	142.0 (101.0–230.0)	110.5 (76.5–176.5)	0.18
Aspartate aminotransferase (UI.L^-1^)	135.0 (49.0–323.0)	124.0 (43.0–284.0)	153.5 (56.5–333.0)	0.08
Alanine aminotransferase (UI.L^-1^)	53.0 (33.0–113.0)	60.0 (37.0–119.0)	51.0 (31.0–111.0)	0.05
During ICU stay				
Epinephrine within 24 hours of admission	26 (22.8%)	18 (31.6%)	8 (14.0%)	0.03
Norepinephrine within 24 hours of admission	62 (54.4%)	31 (54.4%)	31 (54.4%)	1.00
Dobutamine within 24 hours of admission	43 (37.7%)	19 (33.3%)	24 (42.1%)	0.33
Mechanical ventilation within 24 hours of admission	100 (87.7%)	50 (87.7%)	50 (87.7%)	1.00
ECLS	15 (13.2%)	9 (15.8%)	6 (10.5%)	0.41
ECLS Delay after ICU admission (days)	1.0 (0.0–1.0)	1.0 (0.0–1.0)	0.5 (0.0–1.0)	0.69
Central venous catheter	101 (88.6%)	50 (87.7%)	51 (89.5%)	0.77
Dialysis catheter	31 (27.2%)	17 (29.8%)	14 (24.6%)	0.53
Electrical pacing	6 (5.3%)	4 (7.0%)	2 (3.5%)	0.68
Arterial catheter (excluding angiography catheter)	97 (85.1%)	50 (87.7%)	47 (82.5%)	0.43
Intra-aortic balloon pump	17 (14.9%)	11 (19.3%)	6 (10.5%)	0.19
Proton pump inhibitor	108 (94.7%)	55 (96.5%)	53 (93.0%)	0.40
Outcomes during ICU stay				
TIMI major or minor bleeding	47 (41.2%)	12 (21.1%)	35 (61.4%)	<0.0001
Delay after ICU admission (days)	1.0 (0.0–2.0)	0.0 (0.0–1.0)	1.0 (1.0–3.0)	0.009
TIMI major bleeding	27 (23.7%)	7 (12.3%)	20 (35.1%)	0.004
TIMI minor bleeding	20 (17.5%)	5 (8.8%)	15 (26.3%)	0.01
Minimum hemoglobin level (g.dL^-1^)	9.1 (7.7–11.2)	10.2 (8.2–12.0)	8.5 (7.2–10.5)	0.009
Hemoglobin variation since admission (g.dL^-1^)	-2.0 (-4.1 –-0.8)	-1.5 (-2.6 –-0.5)	-3.2 (-5.2 –-1.5)	0.009
Red blood cell transfusion	38 (33.3%)	14 (24.6%)	24 (42.1%)	0.04
Total red blood cell transfusion (units)	0.0 (0.0–2.0)	0.0 (0.0–0.0)	0.0 (0.0–4.0)	0.02
Platelet transfusion	7 (6.1%)	2 (3.5%)	5 (8.8%)	0.24
Total platelet transfusion (units)	0.0 (0.0–0.0)	0.0 (0.0–0.0)	0.0 (0.0–0.0)	0.20
Plasma transfusion	6 (5.3%)	3 (5.3%)	3 (5.3%)	1.0
Total plasma transfusion (units)	0.0 (0.0–0.0)	0.0 (0.0–0.0)	0.0 (0.0–0.0)	0.91
ICU length of stay (days)	4.0 (3.0–8.0)	3.0 (2.0–7.0)	4.0 (3.0–9.0)	0.19
Death in ICU	43 (37.7%)	26 (45.6%)	17 (29.8%)	0.08

Results are expressed as median (first and third quartile) and numbers (proportions). ACS: Acute coronary syndrome; aPTT: Activated partial thromboplastin time; ECLS: Extracorporeal life support; ICU: Intensive Care Unit; NA: Not applicable; STEMI: ST-segment elevation myocardial infarction.

### Outcomes of the 114 matched patients

The primary and secondary outcomes of matched patients treated with ticagrelor and clopidogrel are summarized in **Table 2**. Bleeding was observed in 47 patients (41.2%), with TIMI major bleeding occurring in 27 patients (23,7%) and TIMI minor bleeding occurring in 20 patients (17,5%). Of the 47 patients with bleeding, 12 (21.1%) were treated with clopidogrel and 35 (61.4%) with ticagrelor (*p*< 0.0001). This significant association was found for both TIMI major bleeding (12.3% *vs*. 35.1%, *p* = 0.004) and TIMI minor bleeding (8.8% *vs*. 26.3%, *p* = 0.01).

Hemoglobin levels at ICU admission were not significantly different between the ticagrelor group and the clopidogrel group (12.2 (10.6–13.7) *vs*. 12.4 (10.5–13.7) g.dL^-1^, *p* = 0,89). However, during ICU stay, patients treated with ticagrelor had lower minimal hemoglobin levels (8.5 (7.2–10.5) *vs*. 10.2 (8.2–12.0) g.dL^-1^, *p* = 0.009), a greater variation in hemoglobin levels (-3.2 (-5.2 –-1.5) *vs*. -1.5 (-2.6 –-0.5) g.dL^-1^, *p* = 0.009), and a greater need for red blood cell transfusion (0 (0–4) *vs*. 0 (0–0) units, *p* = 0.02).

Death in ICU occurred in 26 patients (45.6%) treated with clopidogrel and in 17 patients (29.8%) treated with ticagrelor (*p* = 0.08). ICU length of stay was 4.0 (3.0–8.0) days, with no significant difference between the two groups (*p* = 0.19).

### Ticagrelor and bleeding risk

**Table 3** and **Table 4** show the characteristics associated with bleeding before and during ICU. Bleeding during ICU stay was significantly associated with ticagrelor treatment (74.5% *vs*. 32.8%, *p*< 0.0001; relative risk 2.60 (1.55–4.35)), lower left ventricular ejection fraction (30 (20–40) *vs*. 40% (30–50), *p* = 0,002), mechanical ventilation within 24 hours of ICU admission (100.0% *vs*. 79.1%, *p* = 0.0008), central venous catheter (100.0% *vs*. 80.6%, *p* = 0.001), arterial catheter (95.7% *vs*. 77.6%, *p* = 0.007), ECLS (4.5% *vs*. 25.5%, *p* = 0.001), and intra-aortic balloon pump (23.4% *vs*. 9.0%, *p* = 0.03).

**Table 3:** Characteristics associated with bleeding before and during ICU stay.

Variables	Bleeding during ICU stay (TIMI major or minor)		*P value*	Relative risk (CI 95%)
	No (*n* = 67)	Yes (*n* = 47)		
Age (years)	63.0 (54.6–73.1)	66.0 (58.7–73.0)	0.63	-
Male gender	52 (77.6%)	31 (66.0%)	0.17	0.72 (0.40–1.32)
History of				
Hypertension	45 (67.2%)	30 (63.8%)	0.71	0.92 (0.51–1.66)
Diabetes mellitus	42 (62.7%)	24 (51.1%)	0.22	0.76 (0.43–1.34)
Smoking	33 (49.3%)	31 (66.0%)	0.08	1.51 (0.83–2.77)
Dyslipidemia	23 (34.3%)	16 (34.0%)	0.97	0.99 (0.54–1.81)
Chronic heart failure	22 (32.8%)	13 (27.7%)	0.56	0.86 (0.46–1.64)
Coronary artery disease	18 (26.9%)	13 (27.7%)	0.93	1.02 (0.54–1.94)
Peripheral artery occlusive disease	14 (20.9%)	4 (8.5%)	0.07	0.50 (0.18–1.38)
Chronic renal failure	8 (11.9%)	6 (12.8%)	0.89	1.05 (0.44–2.46)
Ischemic stroke	8 (11.9%)	3 (6.4%)	0.52	0.64 (0.20–2.06)
Body mass index > 30kg.m^-2^	8 (11.9%)	3 (6.4%)	0.52	0.64 (0.20–2.06)
Sleep apnea syndrome	4 (6.0%)	4 (8.5%)	0.72	1.23 (0.44–3.43)
Cancer	3 (4.5%)	4 (8.5%)	0.44	1.42 (0.51–3.96)
Chronic obstructive pulmonary disease	3 (4.5%)	4 (8.5%)	0.44	1.42 (0.51–3.96)
Alcoholism	0 (0.0%)	2 (4.3%)	0.17	2.49 (0.60–10.26)
Usual treatment				
Oral anticoagulant	2 (3.0%)	1 (2.1%)	1	0.80 (0.11–5.83)
Aspirin	26 (38.8%)	18 (38.3%)	0.96	0.99 (0.55–1.78)
Ticagrelor	2 (3.0%)	0 (0.0%)	0.51	NA
Clopidogrel	10 (14.9%)	9 (19.1%)	0.55	1.18 (0.57–2.45)
Place of initial care			0.32	
Outside hospital	37 (55.2%)	31 (66.0%)		1.00
In hospital, emergency department	14 (20.9%)	10 (21.2%)		0.91 (0.56–1.51)
In hospital, other department	16 (23.9%)	6 (12.8%)		0.60 (0.29–1.22)
Type of ACS : STEMI*	52 (77.6%)	36 (76.6%)	0,9	0.97 (0.49–1.90)
Cardiac arrest before ICU admission	28 (41.8%)	25 (53.2%)	0.23	1.31 (0.74–2.32)
Initial treatment				
Aspirin	67 (100.0%)	47 (100.0%)	NA	NA
Unfractionated Heparin	12 (17.9%)	8 (17.0%)	0.90	1.00 (0.47–2.15)
Low Molecular Weight Heparin	55 (82.1%)	39 (83.0%)	0.90	1.00 (0.47–2.14)
Glycoprotein IIb/IIIa inhibitors (tirofiban)	19 (28.4%)	17 (36.2%)	0.38	1.20 (0.66–2.19)
Ticagrelor (compared to clopidogrel)	22 (32.8%)	35 (74.5%)	<0.0001	2.60 (1.55–4.35)
Coronarography	62 (92.5%)	46 (97.9%)	0.40	2.57 (0.41–16.1)
Radial approach (compared to femoral approach)	27 (43.5%)	26 (57.8%)	0.15	1.39 (0.77–2.52)
Stent	49 (79.0%)	41 (89.1%)	0.16	1.62 (0.64–4.10)
ECLS during coronarography	1 (1.5%)	4 (8.5%)	0.16	1.99 (0.71–5.56)
At ICU admission				
Simplified Acute Physiology Score II	61.0 (39.0–86.0)	62.0 (44.0–85.0)	0.91	
Therapeutic hypothermia	0 (0.0%)	1 (2.1%)	0.41	2.41 (0.33–17.49)
Left ventricular ejection fraction	0.4 (0.3–0.5)	0.3 (0.2–0.4)	0.002	
Biological parameters at ICU admission				
Hemoglobin level (g.dL^-1^)	12.2 (10.6–13.2)	12.5 (10.5–14.1)	0.2	
Platelet count (G.L^-1^)	227.5 (199.0–276.0)	227.0 (169.0–268.0)	1	
aPTT (ratio)	1.5 (1.3–2.4)	2.2 (1.3–3.7)	0.18	
Prothrombin (%)	67.0 (55.0–76.0)	68.5 (55.0–73.0)	0.7	
Urea (mmol.L^-1^)	7.8 (5.4–14.4)	8.2 (4.8–11.3)	0.63	
Creatinine (μmol.L^-1^)	122.0 (90.0–227.0)	126.0 (79.0–180.0)	0.55	
Aspartate aminotransferase (UI.L^-1^)	108.0 (43.0–84.0)	175.0 (69.0–339.0)	0.91	
Alanine aminotransferase (UI.L^-1^)	52.5 (28.0–119.0)	56.0 (33.0–112.0)	0.59	

Results are expressed as median (first and third quartile) and numbers (proportions). ACS: Acute coronary syndrome; aPTT: Activated partial thromboplastin time; ECLS: Extracorporeal life support; ICU: Intensive Care Unit; NA: Not applicable; STEMI: ST-segment elevation myocardial infarction.

*opposite to “non-ST-segment elevation myocardial infarction”.

**Table 4:** Factors potentially associated with bleeding during ICU stay.

Variables	Total (*n* = 114)	Bleeding during ICU stay (TIMI major or minor)		*P* value
		No (*n* = 67)	Yes (*n* = 47)	
Epinephrine within 24 hours of admission	26 (22.8%)	17 (25.4%)	9 (19.1%)	0.44
Norepinephrine within 24 hours of admission	62 (54.4%)	33 (49.3%)	29 (61.7%)	0.19
Dobutamine within 24 hours of admission	43 (37.7%)	22 (32.8%)	21 (44.7%)	0.2
Mechanical ventilation within 24 hours of admission	100 (87.7%)	53 (79.1%)	47 (100.0%)	0.0008
ECLS	15 (13.2%)	3 (4.5%)	12 (25.5%)	0.001
ECLS delay after ICU admission (days)	1.0 (0.0–1.0)	0.0 (0.0–1.0)	1.0 (0.0–1.0)	0.42
Central venous catheter	101 (88.6%)	54 (80.6%)	47 (100.0%)	0.001
Dialysis catheter	31 (27.2%)	18 (26.9%)	13 (27.7%)	0.93
Electrical pacing	6 (5.3%)	3 (4.5%)	3 (6.4%)	0.69
Arterial catheter (excluding angiography catheter)	97 (85.1%)	52 (77.6%)	45 (95.7%)	0.007
Intra-aortic balloon pump	17 (14.9%)	6 (9.0%)	11 (23.4%)	0.03
Proton pump inhibitor	108 (94.7%)	61 (91.0%)	47 (100.0%)	0.04
Outcomes during ICU stay				
Minimum hemoglobin level (g.dL^-1^)	9.1 (7.7–11.2)	10.7 (8.8–12.0)	8.0 (7.1–9.1)	<0.0001
Hemoglobin variation since admission (g.dL^-1^)	-2.0 (-4.1 –-0.8)	-1.3 (-2.1 –-0.4)	-4.4 (-6.1 –-3.1)	<0.0001
Red blood cell transfusion	38 (33.3%)	8 (11.3%)	30 (63.3%)	< 0.0001
Total red blood cell transfusion (units)	0.0 (0.0–2.0)	0.0 (0.0–0.0)	2.0 (0.0–6.0)	<0.0001
Platelet transfusion	7 (6.1%)	0 (0.0%)	7 (14.9%)	0.001
Total platelet transfusion (units)	0.0 (0.0–0.0)	0.0 (0.0–0.0)	0.0 (0.0–0.0)	0.05
Plasma transfusion	6 (5.3%)	0 (0.0%)	6 (12.8%)	0.004
Total plasma transfusion (units)	0.0 (0.0–0.0)	0.0 (0.0–0.0)	0.0 (0.0–0.0)	0.05
ICU length of stay (days)	4.0 (3.0–8.0)	3.0 (1.0–5.0)	8.0 (4.0–15.0)	<0.0001
Death in ICU	43 (37.7%)	26 (38.8%)	17 (36.2%)	0.78

Results are expressed as median (first and third quartile) and numbers (proportions). ECLS: Extracorporeal life support; ICU: Intensive Care Unit.

While bleeding during ICU stay was significantly associated with greater ICU length of stay (8.0 (4.0–15.0) *vs*. 3.0 (1.0–5.0) days, *p*< 0.0001), it was not significantly associated with ICU mortality (36.2% *vs*. 38.8, *p* = 0.78).

## Discussion

To our knowledge, this is the first study to specifically compare the bleeding risk of ticagrelor and clopidogrel in ICU patients with ACS. A study prior to ours compared the bleeding risk on prasugrel and clopidogrel in cardiogenic shock patients undergoing primary PCI for acute myocardial infarction. The bleeding defined by combined TIMI bleedings at 30-days occurred in 52% on prasugrel group and in 37.1% on clopidogrel group (*p* = 0.09) [19]. A second study, consisting of a post hoc analysis, focused on the comparison of patients on clopidogrel *vs*. ticagrelor or prasugrel in patients with acute myocardial infarction complicated by cardiogenic shock. No significant difference was found regarding the secondary endpoint for bleeding events at 12-months. Unfortunately, in this study, only 18 patients treated with ticagrelor were included (and 93 patients treated with prasugrel); the follow-up was over a year and did not focus on the specific period of stay in ICU, as in our study [20].

Many studies of large cohorts conducted outside the ICU have reported an increased bleeding risk of ticagrelor compared to clopidogrel, despite an overall improvement in mortality [2,3]. The PLATO study, in which patients were followed for a period of 13 months, found that the superiority of ticagrelor in cardiovascular events was apparent within 30 days of treatment initiation and persisted throughout the study period, whereas most bleeding occurred after 30 days of treatment [3]. In our study of critically ill patients with severe forms of ACS, all bleeding events were observed early in the study, the median ICU length of stay being 4 (3–8) days.

A post-hoc study using data from the PLATO cohort subdivided the groups of patients treated with ticagrelor and clopidogrel according to the Killip classification (previous Heart Failure (HF) *vs*. clinical signs of HF on admission (Killip class II and III) *vs*. no HF (Killip class I) [21]. Patients in the ticagrelor group were found to have a lower risk of composite ischemic endpoint than patients in the clopidogrel group, regardless of HF status (hazard ratio (HR) 0.87 (95% CI: 0.73–1.03) in patients with HF and HR 0.84 (95% CI: 0.75–0.93) in patients with no HF, *p* = 0 .76). Moreover, no differences in major bleeding were observed between the two groups, again regardless of HF status (HR 1.08 (95% CI: 0.87–1.34)) [22]. The difference between these and our results may be explained by the fact that Killip class IV patients (*i*.*e*., cardiogenic shock—hypotension (systolic < 90 mm Hg) with signs of peripheral vasoconstriction (oliguria, cyanosis, sweating)) were excluded from this study and not in ours. Our patients were more critically ill and, consequently, required intensive care which put them at a much higher risk of bleeding.

A retrospective study of 81 cardiac surgery patients reported an increased risk of bleeding in patients treated with aspirin and ticagrelor compared to patients treated with aspirin and clopidogrel [23]. This study also found greater perioperative blood loss, an increased use of blood components, and more frequent recourse to surgical revision for postoperative bleeding in patients treated with ticagrelor. Coronary artery bypass grafting is sometimes urgently required in the initial care of ACS. In our study, we excluded those patients, to focus on the non-CABG related bleeding risk in ICU. Nevertheless, those patients may require intensive care, and the bleeding risk of a dual antiplatelet therapy by aspirin and ticagrelor in this context is yet to be determined.

The management of severe bleeding in patients treated with ticagrelor remains challenging. Platelet transfusion, which is normally performed to reverse the effects of antiplatelet drugs, has been found to be inefficient in the first 24 hours in ticagrelor-treated patients, likely because circulating ticagrelor and its active metabolite (whose half-lives are 9 and 12 hours, respectively) inhibit fresh platelets [24–27]. A specific antidote to ticagrelor is currently being researched to address this problem [28]. Finally, the transition from clopidogrel to ticagrelor is the only switch between P2Y12 inhibitors that has been investigated in a trial powered for clinical endpoint; efficacy and safety of ticagrelor were not affected by previous clopidogrel exposure [2]. While many studies concerning switching from ticagrelor to clopidogrel have been conducted, this practice is discouraged due to a lack of safety/efficacy data [4,29–31].

Our study focused on the intensive care management of 155 ICU patients diagnosed with severe forms of ACS. After propensity score matching, 57 patients treated with ticagrelor were compared to 57 patients treated with clopidogrel. All of our study patients were critically ill, as evidenced by the SAPS II (61.5 (41.0–85.0)) and by other indicators such as the rate of cardiac arrest before ICU admission (46.5%) or the use of ECLS during ICU stay (13.2%). As expected, intensive care management of these patients required the use of invasive techniques: for example, a central venous catheter was inserted in almost 90% of patients and a dialysis catheter in more than 25% of patients.

Our analysis of the primary outcome yielded interesting results. The first was the high occurrence of bleeding events (64%), which was estimated using a clinically relevant definition of major and minor bleeding derived from the TIMI classification. This finding was confirmed by the significant variation in hemoglobin levels and the significant use of red blood cell transfusion. In fact, bleeding complications are a frequent and serious occurrence in ICU. Accordingly, the occurrence of TIMI major and minor bleeding in our study was 61.4% and 21.0%, respectively, in marked contrast with the results of the PLATO study, in which the occurrence of TIMI major and minor bleeding was 11.1% for the ticagrelor group and 10.9% for the clopidogrel group.

Another interesting finding of our study was that in the ICU setting, the relative risk of bleeding occurrence in ACS patients treated with ticagrelor compared to clopidogrel is very high (HR 2.60 (1.55–4.35)). In fact, of all the parameters studied, only invasive medical techniques (*i*.*e*., ECLS, venous and arterial catheter, and mechanical ventilation) were significantly associated with bleeding. While this finding is not new, it reinforces the observation that bleeding is more prevalent in ICU.

Our analysis of secondary outcomes also yielded interesting results. As regards ICU mortality, death occurred in 26 (45.6%) patients treated with clopidogrel *vs*. 17 (29.8%) patients treated with ticagrelor—a difference that was not statistically significant (*p* = 0.08). Given the risk of serious bleeding associated with ticagrelor, we had expected this treatment to be associated with increased mortality. Yet, not only was this not the case, but we actually found the opposite tendency. Two hypotheses can be made to explain this finding. The first is that the patients treated with clopidogrel were more critically ill than those treated with ticagrelor, the SAPS II being 69.0 (46.0–88.0) for the clopidogrel group and 60.0 (38.0–80.0) for the ticagrelor group (*p* = 0.03). It should be recalled that the SAPS II was not included in the propensity score: the former is calculated in the first 24 hours in ICU, whereas the latter relates to the period preceding treatment selection. The second hypothesis is that the increased bleeding risk associated with ticagrelor is offset by the early cardiovascular benefits of this treatment; the wide inter individual variability in the pharmacodynamic response to clopidogrel, linked to several factors, including genotype polymorphisms, could support this hypothesis [32,33]. We cannot conclude on this, however, because while it may be true that ticagrelor is more effective than clopidogrel (as frequently reported in the literature), the relative efficacy of ticagrelor was not the focus of our study [2].

Our study has several limitations. The first limitation is the retrospective and monocentric nature of the study, which prevented us from concluding on the existence of a causal link between ticagrelor and increased bleeding risk. However, our use of a high-level methodology, lends robustness to our results. It should nevertheless be noted that propensity score matching may have led us to omit some of the factors related to the initial choice of antiplatelet therapy.

Finally, our study cannot suggest a contraindication to ticagrelor in the management of ICU patients with ACS. Our analysis focused on bleeding complications in the initial phase of ACS management, when in fact the final choice between clopidogrel and ticagrelor must be made based on long-term evaluation of treatment efficacy. There is a major lack of prospective randomized study in this sub-category of patient with a severe form of ACS.

## Conclusions

Bleeding complications are frequent and serious in ICU patients with ACS. A dual antiplatelet therapy of aspirin and ticagrelor is associated with a higher risk of bleeding compared to a dual antiplatelet therapy of aspirin and clopidogrel. More studies on this topic are urgently needed to help improve the intensive care management of patients with severe forms of ACS.

## Supporting information

S1 Table Characteristics of the 155 included patients at study inclusion. (DOC)

S2 Table Characteristics and outcomes of the 155 included patients between study inclusion and Intensive Care Unit discharge or death. (DOC)
