# Supplementary material for: Bleeding risk of ticagrelor compared to clopidogrel in intensive care unit patients with acute coronary syndrome: A propensity-score matching analysis
Source: PLoS One. 2020 May 4;15(5):e0232768. doi: 10.1371/journal.pone.0232768 (PMC7197796; doi:10.1371/journal.pone.0232768)
Supplement: S1 Table — (DOC) [file pone.0232768.s001.doc]

**Table S1:** Characteristics of the 155 included patients at study inclusion.

| **Variables** | **All patients**  **(*n*=155)** | **Clopidogrel**  **(*n*=73)** | **Ticagrelor**  **(*n*=82)** | **Standardized difference** |
| --- | --- | --- | --- | --- |
| Age (years) | 65.0 (55.8 – 73.0) | 67.4 (61.0 – 73.6) | 61.8 (54.1 – 71.5) | -0.39 |
| Male gender | 112 (72.3%) | 53 (72.6%) | 59 (72.0%) | -0.01 |
| History of |  |  |  |  |
| Hypertension | 100 (64.5%) | 54 (74.0%) | 46 (56.1%) | -0.38 |
| Diabetes mellitus | 93 (60.0%) | 49 (67.1%) | 44 (53.7%) | -0.28 |
| Smoking | 89 (57.4%) | 38 (52.1%) | 51 (62.2%) | 0.20 |
| Dyslipidemia | 53 (34.2%) | 30 (41.1%) | 23 (28.0%) | -0.28 |
| Chronic heart failure | 46 (29.7%) | 26 (35.6%) | 20 (24.4%) | -0.25 |
| Coronary artery disease | 42 (27.1%) | 23 (31.5%) | 19 (23.2%) | -0.19 |
| Chronic renal failure | 27 (17.4%) | 20 (27.4%) | 7 (8.5%) | -0.50 |
| Peripheral artery occlusive disease | 24 (15.5%) | 12 (16.4%) | 12 (14.6%) | -0.05 |
| Body mass index > 30kg.m^-2^ | 16 (10.3%) | 9 (12.3%) | 7 (8.5%) | -0.12 |
| Ischemic stroke | 15 (9.7%) | 9 (12.3%) | 6 (7.3%) | -0.17 |
| Chronic obstructive pulmonary disease | 13 (8.4%) | 5 (6.8%) | 8 (9.8%) | 0.10 |
| Sleep apnea syndrome | 9 (5.8%) | 3 (4.1%) | 6 (7.3%) | 0.14 |
| Cancer | 7 (4.5%) | 4 (5.5%) | 3 (3.7%) | -0.09 |
| Hazardous alcohol use | 3 (1.9%) | 2 (2.7%) | 1 (1.2%) | -0.11 |
| Usual treatment |  |  |  |  |
| Oral anticoagulant | 3 (1.9%) | 2 (2.7%) | 1 (1.2%) | -0.11 |
| Aspirin | 58 (37.4%) | 28 (38.4%) | 30 (36.6%) | -0.04 |
| Clopidogrel | 27 (17.4%) | 18 (24.7%) | 9 (11.0%) | -0.36 |
| Ticagrelor | 3 (1.9%) | 2 (2.7%) | 1 (1.2%) | -0.11 |
| Place of initial care |  |  |  | -0.08 |
| Outside hospital | 79 (51.0%) | 39 (53.4%) | 40 (48.8%) |  |
| In hospital, emergency department | 49 (31.6%) | 17 (23.3%) | 32 (39.0%) |  |
| In hospital, other department | 27 (17.4%) | 17 (23.3%) | 10 (12.2%) |  |
| Cardiac arrest before ICU admission | 63 (40.6%) | 32 (43.8%) | 31 (37.8%) | -0.12 |
| Type of ACS : STEMI | 120 (77.4%) | 53 (72.6%) | 67 (81.7%) | 0.22 |

Results are expressed as median (first and third quartile) and numbers (proportions). ACS: Acute coronary syndrome; ICU: Intensive Care Unit; STEMI: ST-segment elevation myocardial infarction.
