# Supplementary material for: Bleeding risk of ticagrelor compared to clopidogrel in intensive care unit patients with acute coronary syndrome: A propensity-score matching analysis
Source: PLoS One. 2020 May 4;15(5):e0232768. doi: 10.1371/journal.pone.0232768 (PMC7197796; doi:10.1371/journal.pone.0232768)
Supplement: S2 Table — (DOC) [file pone.0232768.s002.doc]

**Table S2:** Characteristics and outcomes of the 155 included patients between study inclusion and Intensive Care Unit discharge or death.

| **Variables** | **All patients**  **(*n*=155)** | **Clopidogrel**  **(*n*=73)** | **Ticagrelor**  **(*n*=82)** | ***P* value** |
| --- | --- | --- | --- | --- |
| Coronary angiography | 147 (94.8%) | 66 (90.4%) | 81 (98.8%) | 0.03 |
| Stent | 116 (78.9%) | 51 (77.3%) | 65 (80.2%) | 0.66 |
| ECLS during coronary angiography | 6 (3.9%) | 4 (5.5%) | 2 (2.4%) | 0.42 |
| Initial treatment |  |  |  |  |
| Glycoprotein IIb/IIIa inhibitors (tirofiban) | 48 (31.0%) | 16 (21.9%) | 32 (39.0%) | 0.02 |
| Aspirin | 155 (100.0%) | 73 (100.0%) | 82 (100.0%) | NC |
| Unfractionated Heparin | 128 (82.6%) | 55 (75.3%) | 73 (89.0%) | 0.02 |
| Low Molecular Weight Heparin | 27 (17.4%) | 18 (24.7%) | 9 (11.0%) | 0.02 |
| At ICU admission |  |  |  |  |
| Simplified Acute Physiology Score II | 60.0 (38.0 – 83.0) | 69.0 (50.0 – 86.0) | 52.5 (35.0 – 72.0) | 0.0003 |
| Therapeutic hypothermia | 1 (0.6%) | 0 (0.0%) | 1 (1.2%) | 1 |
| Left ventricular ejection fraction | 0.3 (0.2 – 0.5) | 0.3 (0.2 – 0.5) | 0.4 (0.3 – 0.5) | 0.51 |
| Biological parameters at ICU admission |  |  |  |  |
| Hemoglobin level (g.dL^-1^) | 12.3 (10.5 – 13.8) | 12.2 (10.3 – 13.6) | 12.5 (10.8 – 14.0) | 0.13 |
| Platelet count (G.L^-1^) | 228.0 (181.0 – 276.0) | 218.0 (171.0 – 268.0) | 239.5 (188.5 – 291.0) | 0.19 |
| aPTT ratio | 1.9 (1.3 – 3.7) | 1.6 (1.3 – 4.2) | 2.0 (1.2 – 3.7) | 0.97 |
| Prothrombin (%) | 68.0 (55.0 – 76.0) | 67.0 (55.0 – 76.0) | 68.0 (55.0 –76.0) | 0.34 |
| Urea (mmol.L^-1^) | 8.2 (5.4 – 14.4) | 10.6 (6.2 – 15.6) | 7.1 (4.9 – 10.2) | 0.03 |
| Creatinine (μmol.L^-1^) | 124.0 (86.0 – 213.0) | 155.0 (112.0 – 260.0) | 105.0 (78.0 – 154.0) | 0.002 |
| Aspartate aminotransferase (UI.mL^-1^) | 130.0 (49.0 – 323.0) | 124.0 (49.0 – 284.0) | 140.0 (51.0 – 328.0) | 0.27 |
| Alanine aminotransferase (UI.mL^-1^) | 55.5 (26.0 – 119.0) | 61.0 (28.0 – 136.0) | 53.0 (23.0 – 112.0) | 0.09 |
| During ICU stay |  |  |  |  |
| Epinephrine within 24 hours of admission | 33 (21.3%) | 23 (31.5%) | 10 (12.2%) | 0.003 |
| Norepinephrine within 24 hours of admission | 80 (51.6%) | 40 (54.8%) | 40 (48.8%) | 0.45 |
| Dobutamine within 24 hours of admission | 54 (34.8%) | 23 (31.5%) | 31 (37.8%) | 0.41 |
| Mechanical ventilation within 24 hours of admission | 133 (85.8%) | 64 (87.7%) | 69 (84.1%) | 0.53 |
| ECLS | 21 (13.5%) | 10 (13.7%) | 11 (13.4%) | 0.96 |
| ECLS delay after ICU admission (days) | 1.0 (0.0 – 1.0) | 0.5 (0.0 – 1.0) | 1.0 (0.0 – 1.0) | 0.49 |
| Central venous catheter | 82 (52.9%) | 63 (86.3%) | 67 (81.7%) | 0.44 |
| Dialysis catheter | 38 (24.7%) | 21 (28.8%) | 17 (21.0%) | 0.26 |
| Electrical pacing | 7 (4.5%) | 4 (5.5%) | 3 (3.7%) | 0.59 |
| Arterial catheter (excluding angiography catheter) | 127 (81.8%) | 62 (84.9%) | 65 (79.3%) | 0.36 |
| Intra-aortic balloon pump | 21(13.5%) | 11 (15.1%) | 10 (12.2%) | 0.60 |
| Proton pump inhibitor | 147 (95.4%) | 71 (97.3%) | 76 (93.8%) | 0.31 |
| Outcomes during ICU stay |  |  |  |  |
| TIMI major or minor bleeding | 64 (41.3%) | 14 (19.2%) | 50 (61.0%) | <0.0001 |
| Delay after ICU admission (days) | 1.0 (0.0 – 2.0) | 1.0 (0.0 – 2.0) | 1.0 (1.0 – 2.0) | 0.48 |
| TIMI major bleeding | 39 (25.2%) | 8 (10.9%) | 31 (37.8%) | 0.0001 |
| TIMI minor bleeding | 25 (16.1%) | 6 (8.2%) | 19 (23.2%) | 0.01 |
| Minimum hemoglobin level (g.dL^-1^) | 9.3 (7.8 – 11.4) | 9.8 (8.3 – 11.9) | 8.5 (7.3 – 10.6) | 0.01 |
| Hemoglobin variation since admission (g.dL^-1^) | -1.9 (-4.1 – -0.8) | -1.3 (-2.2 – -0.5) | -3.1 (-5.1 – -1.4) | 0.0002 |
| Red blood cell transfusion | 52 (33.5%) | 17 (23.3%) | 35 (42.7%) | 0.01 |
| Total red blood cell transfusion (units) | 0.0 (0.0 – 2.0) | 0.0 (0.0 – 0.0) | 0.0 (0.0 – 4.0) | 0.008 |
| Platelet transfusion | 8 (5.2%) | 2 (2.7%) | 6 (7.3%) | 0.20 |
| Total platelet transfusion (units) | 0.0 (0.0 – 0.0) | 0.0 (0.0 – 0.0) | 0.0 (0.0 – 0.0) | 0.21 |
| Plasma transfusion | 6 (3.9%) | 3 (4.1%) | 3 (3.7%) | 0.88 |
| Total plasma transfusion (units) | 0.0 (0.0 – 0.0) | 0.0 (0.0 – 0.0) | 0.0 (0.0 – 0.0) | 0.98 |
| ICU length of stay (days) | 4.0 (2.0 – 8.0) | 3.0 (2.0 – 7.0) | 4.0 (3.0 – 10.0) | 0.05 |
| Death in ICU | 58 (37.4%) | 34 (46.6%) | 24 (29.3%) | 0.03 |

Results are expressed as median (first and third quartile) and numbers (proportions). aPTT: Activated partial thromboplastin time; ECLS: Extracorporeal life support; ICU: Intensive Care Unit.
